# Exploration of production conditions: a step towards the development of a community-based breeding program for Butana cattle

**DOI:** 10.1007/s11250-020-02459-4

**Published:** 2020-11-17

**Authors:** Elhady A. M. Omer, Sowah Addo, Regina Roessler, Jonas Schäler, Dirk Hinrichs

**Affiliations:** 1 Department of Organic Agricultural Sciences, University of Kassel, 37213 Witzenhausen, Germany; 2 Department of Animal breeding and Genetics, University of Khartoum, Khartoum, Sudan

**Keywords:** Butana cattle, Breed development, Breeding objectives

## Abstract

In Sudan, many Butana cattle farmers practice indiscriminate crossbreeding to improve the milk yield performance of cows, as organized breeding programs are lacking. Objectives of this study were to identify the current production conditions of Butana cattle and to determine farmers’ production objectives and trait preferences using a field survey. The overall aim was to explore the possibility of establishing a community-based breeding program for the genetic improvement of the breed. A semi-structured questionnaire and field visits were used to collect data from 202 Butana cattle owners. Data were analyzed using chi-squared test, multiple response analysis, and binary logistic regression. Our results showed that Butana cattle farmers mainly raised their animals for milk production. On a five-point scale (5 = most important), milk yield (4.6 ± 0.05), growth performance (4.0 ± 0.07), and lactation length (3.9 ± 0.08) were highly preferred for future development of the breed. One-third of the farmers kept crossbred cattle with on average 4 crossbred animals per herd. About two-thirds of respondents were willing to adopt crossbreeding using exotic breeds to increase milk performance and about the same proportion were willing to exchange breeding bulls and establish farmers’ associations. None of the respondents kept written performance records. However, educated farmers were more likely to adopt record keeping. Farmers’ willingness to engage in associations could be useful for the establishment of a community-based breeding program. Based on the current farmers’ production objectives, the future breeding program should emphasize increasing milk production of the Butana cattle by using improved Butana bulls in village herds.

## Introduction

Indigenous livestock contribute to milk and meat supply, and represent an essential source of employment, income creation, and export earnings of many communities in rural areas in African countries (FAO 2015; Behnke and Osman 2016). In Sudan, the number of indigenous cattle is estimated at 31 million heads (FAO 2019). They are kept under different production systems, e.g., mobile, sedentary, pastoral, and agro-pastoral production. Among indigenous Sudanese cattle, the Butana breed is considered to be one of the most promising breeds suited for milk production in semi-arid regions (Musa et al. 2005; Badri et al. 2011). It plays an essential role in milk supply in addition to fulfilling other functions such as the provision of draught power, insurance, and socio-cultural needs of rural communities. Under improved management conditions, Butana cattle are able to produce more than 1500 kg of milk per lactation (Ahmed et al. 2007); however, they produce less milk under low input farm conditions (Musa et al. 2005). Generally, the traditional production systems under which Butana cattle as well as other indigenous breeds are managed do not match the increasing demands for milk in urban areas (Ahmed et al. 2007). Consequently, crossbreeding with exotic breeds is practiced indiscriminately by smallholder producers without any formal breeding policy or breeding programs to conserve the breed (Musa et al. 2008).

As early as 1945, the Sudan government began a within-breed selection improvement program for Butana cattle by establishing the Atbara Livestock Research Station (Saeed et al. 1987). However, the initiative was met with a series of problems associated with inadequate financial support, and the lack of infrastructure, managerial, and technical skills. To our knowledge, Butana cattle owners were not actively involved in the government’s instituted breed improvement program. The poor involvement of livestock producers in the design and implementation of breeding programs is one of several factors that affect the development of indigenous livestock breeds in developing countries (Philipsson et al. 2011; Wurzinger et al. 2011).

The aims of the current study were to explore the production conditions of Butana cattle and to identify farmers’ production objectives and trait preferences as a step towards the development of a community-based breeding program for the breed. Moreover, breeding and husbandry management, production constraints, and future development aspects were evaluated.

## Materials and methods

### Study location

The study was conducted in the Butana region of central Sudan, between River Nile, Atbara River, and Blue Nile, at latitude 14° 23′ and 17° 34′ N, longitude 32°32′ and 35°36′ E, and at an altitude 345 m above sea level. The region is semi-arid and characterized by high temperatures reaching over 38.5 °C. Annual rainfall amounts to about 300 mm while the dry season extends to around 8 months (Bahbahani et al. 2018).

### Data collection

The data comprised a sample of 202 semi-structured questionnaires answered by owners of Butana cattle randomly selected from 17 villages in the study area. The villages were selected based on the clustering of Butana cattle owners under the guidance of Atbara livestock research station staff. Interviews were carried out from October 2018 to January 2019. The semi-structured questionnaire covered (1) socio-economic characteristics; (2) herd sizes and composition; (3) production objectives; (4) feeding management; (5) animal health management; (6) breeding management; (7) farm income and milk marketing; (8) farmers’ evaluation of adaptation and production traits; (9) production challenges; and (10) future development aspects. Based on a five-point scale (from 1 = very poor to 5 = very good), respondents were asked to evaluate the performance of Butana cattle for milk production, lactation length, growth rate, disease tolerance, and grazing ability (being able to walk long distances in search of pastures and watering points). The respondents were also asked to score their preferences for the traits’ improvement in the future on a scale of five points (from 1 = not important to 5 = most important). On the same basis, respondents were asked to score five major challenges of cattle production including high costs of concentrate feeds, lack of financial supports, lack of cattle improvement services, scarcity of rangeland, and competition from crossbred dairy cattle in terms of milk yield and selling price. The selection of these challenges was motivated by reports of previous studies, e.g., Musa et al. (2006) and Ahmed et al. (2007). Additionally, three separate group discussions involving 4–11 herd owners were carried out to elicit information about production challenges that were not listed on the questionnaires. These group discussions, together with direct observations made during the survey, enhanced the data collected.

### Statistical analyses

The collected data were analyzed using the statistical package for social sciences (SPSS) version 20 (IBM SPSS 2011) and the results were graphically presented using R software version 3.6.3 (R Core Team 2020). Descriptive statistic measures, which include means and their standard deviation, frequencies, and percentages, and furthermore, statistical tests including chi-squared test for categorical variables and multiple response analysis, were employed. As a form of post hoc analysis, the standardized residuals of the chi-squared results were analyzed applying Bonferroni correction and by the use of the R package “chisq.posthoc.test” (Ebbert 2019). To test the effect of farmers’ education level on their willingness to participate in/perform practices associated with the future development of the breed (i.e., information exchange, adoption of crossbreeding, exchange of breeding bulls, participation in farmers’ associations, and keeping records), a binary logistic regression model was applied. Related to the binary logistic regression test, the levels of education of the farmers were rearranged into two groups: low education consisting of informal and primary education, and high education that consisted of secondary and graduate studies. These two groups were used as predictor variables, while the five different practices were used as response variables.

## Results

### Socio-economic characteristics

**Fig. 1:**
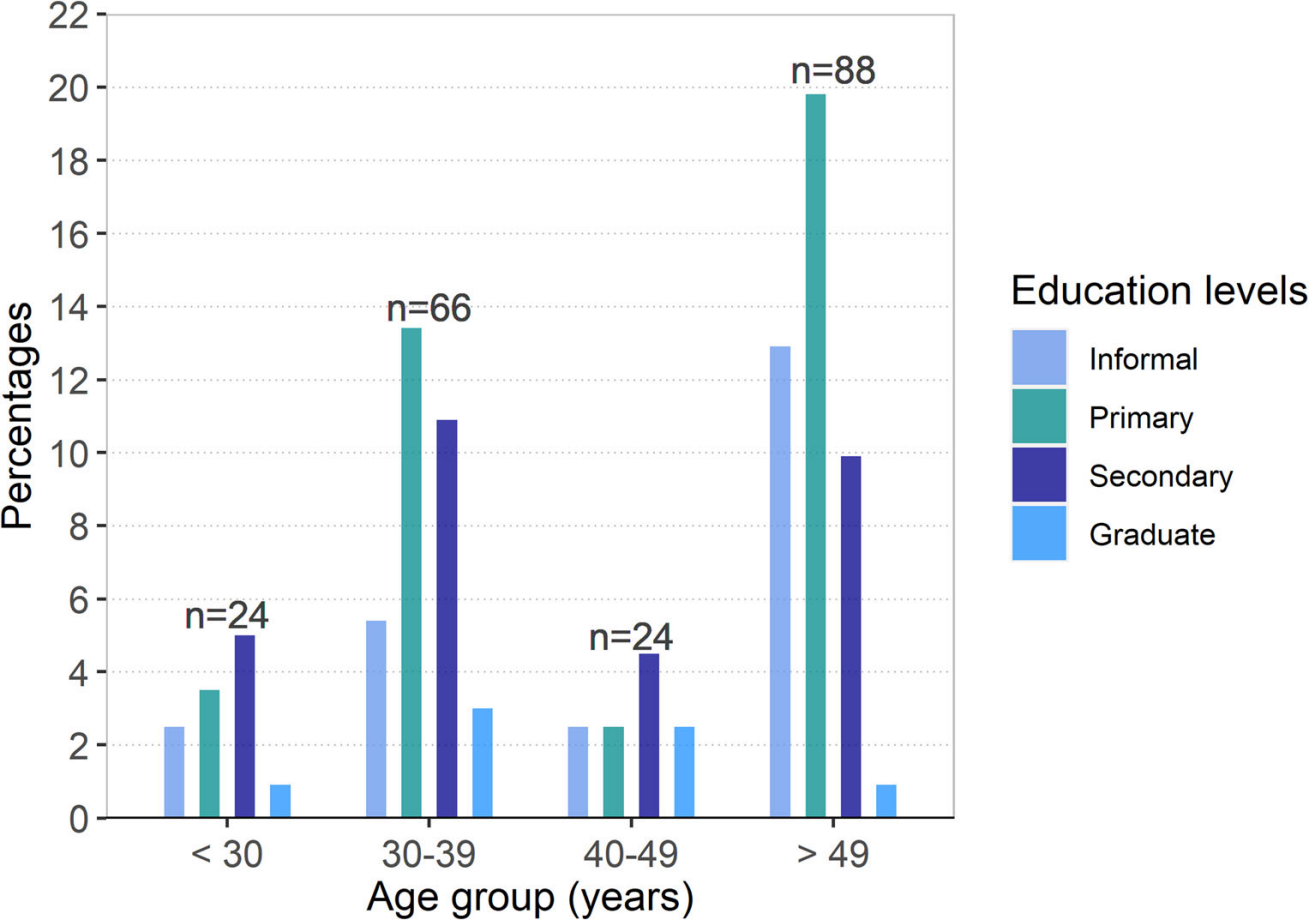
Educational level of farmers of different age groups

All household heads interviewed were men. The majority were above 49 years (43.5%) or between 30 and 39 years (32.7%) old, while 11.9% were between 40 and 49 or below 30 years old, respectively. Primary education was predominant among older (> 49 years) and middle age (30–39 years) farmers and the former had a high proportion of individuals who had only informal education (Fig. 1). Secondary education was predominant among young farmers (< 30 years old) and those between 40 and 49 years of age, while across all age groups, only a few farmers were graduates from higher education. The majority of respondents (59.9%) owned the farming land and almost all of them (89.6%) cultivated crops including sorghum, fruits, vegetables, groundnuts, and dates for household consumption and for sale to generate cash income (Table 1). Farmers use only a small part of their farmland for livestock fodder cultivation, and for landless farmers, land is rented for crop cultivation and for grazing their cattle. Regarding the responsibilities of the family members and labor involvement in the management of cattle herds, 83.7% of the household heads (men) indicated they were the main individuals responsible for farm decision-making, e.g., product marketing, selection of replacement animals, and the culling of cattle. About 40.1% and 47.6% of the respondents relied on their boys and hired laborers for cattle herding, respectively. Only 2% of women were involved in cattle management, mainly milk processing for home consumption or the collection of grasses from the fields.

**Table 1:** Households’ socio-economic characteristics

Categorized variables	*n*	%
Type of land tenure		
Own land	121	59.9
Landless	81	40.1
Activities beside cattle raising		
Crop farming	181	89.6
Other	21	10.4
Family members’ involvement in cattle farming^a^		
Men	169	83.7
Women	4	2.0
Boys	83	41.1
Laborer’s employment		
Hired	96	47.5

^a^More than one answer allowed for

*n*, number of respondents

### Herd composition and production characteristics of Butana cattle

The majority of farmers (65.3%) owned between 1 and 11 cattle heads, and the proportions were 18.4% and 16.3% for farmers who owned 12 to 20 heads, and above 20 heads, respectively. There was a significant relationship between herd size and the employment of laborers on the farm (*χ*^2^ = 10.74, *p* = 0.005). There was no significant association between herd size and farmer’s age (*χ*^2^ = 10.38, *p* = 0.11). On average, cattle herds were composed of 8.2 ± 0.70 cows and 1.2 ± 0.04 breeding bulls (Table 2). According to farmers’ recall, the average daily milk yield per cow of the Butana breed was 6.6 ± 0.38 kg. Lactation length and calving interval were about 6.7 ± 0.10 months and 13.6 ± 0.20 months, respectively (Table 2). The farmers keeping crossbred cattle (*n* = 61) had on average 3.8 ± 0.65 animals per herd. Farmers were not asked to provide information about the level of exotic blood of their crossbred cattle.

**Table 2:** Herds composition and production characteristics of Butana cattle

Items	n	Mean	SE
Herd composition			
Cows	202	8.2	0.70
Heifer and calves	202	4.1	0.29
Bulls	121	1.2	0.04
Oxen	24	1.9	0.20
Overall	202	12.3	0.77
Production characteristics			
Milk yield (kg/day)	202	6.6	0.38
Lactation length (months)	202	6.7	0.10
Calving interval (months)	202	13.6	0.20

*n*, number of herds; *SE*, standard error of the mean

## Production objectives

All farmers stated that they kept Butana cattle mainly for milk production, either for home consumption or to be sold for cash income. A considerable number of the farmers (42%) kept cattle for meat production, meaning that they sold live cattle for slaughter. Only 9% and 8% of the farmers used cattle for draught power (i.e., to prepare the field for crop production) and as an insurance against financial difficulties, respectively. We observed that keeping cattle for draught power was more important for farmers found in areas close to riverbanks, representing about 7% of the respondents.

### Feeding management

The majority of the respondents (75.2%) fed their cattle irrigated fodders. Irrigated fodders comprised *Clitoria* (*Clitoria ternatea*), Abu-70 (*Sorghum bicolor*), or alfalfa (*Medicago sativa*) fed as green or dry. In addition, 65.8% of the farmers offered their cattle concentrate feeds, which were primarily composed of oilseed cakes of sesame (*Sesame indicum*) or groundnuts (*Arachis hypogaea* L.), wheat (*Triticum* L.), bran, sorghum grain, sugar cane (*Saccharum officinarum*), molasses, and cotton (*Gossypium hirsutum*) seed cake. According to farmers, the concentrate feeds were usually mixed on the farm from all or some of these ingredients, but farmers also purchased concentrate feeds from the markets. The concentrate feeds were usually provided to lactating cows during milking period and to crossbred animals. The provision of concentrate feeds was significantly associated (*χ*^2^ = 5.96, *p* = 0.02) with the type of roughages offered to cattle (Table 3). Farmers who fed their cattle an irrigated green fodder tended to leave out concentrate feeds. The provision of concentrate feeds was not significantly associated with herd size (*χ*^2^ = 5.52, *p* = 0.06). Only 24.8% of the respondents fed their cattle crop residues as supplement mainly in the dry season. There was no significant association between the types of roughages offered to cattle and farmer’s age (*χ*^2^ = 2.5, *p* = 0.48). The crop residues comprised stubble of sugar cane and sorghum or groundnut hulls. The majority (62.4%) of the farmers reported moving their cattle out of the permanent homes to communal grazing areas during the wet season that spans between July and October in order to access green pastures freely. During the dry season (from November up to June), cattle are returned back to the permanent homes, where they graze on rangelands close to riverbanks. In this season, cattle are supplied with roughage fodder (irrigated or crop residues) and concentrates.

**Table 3:** Use of concentrate feeds across type of roughage and herd size

	Concentrate feed provided (%)	Chi-square (*p* value)
Type of roughage		
Crop residues	80	*χ*2 = 5.96 (*p* = 0.02)
Irrigated fodder	61	
Herd sizes (head)		
1–11	64	*χ*2 = 5.52 (*p* = 0.06)
12–20	57	
>20	81	

## Animal health management

According to farmers, private veterinarians provided veterinary services. The majority of respondents (79%) reported that they had limited access to appropriate animal health services, whereas only 21% were satisfied with the animal health services. The latter were specifically those farmers who lived in urban areas. During the survey, we observed that in case of disease occurrence, farmers brought a veterinarian to the farm to diagnose the disease, and to provide treatment for the animal. The most common diseases that farmers experienced on their farms included foot and mouth disease (31%), contagious bovine pleuropneumonia (14%), jaundice disease (14%), mastitis (11%), cowpox (10%), heart water (9%), bovine ephemeral fever (6%), and trypanosomiasis (5%). Farmers mentioned the local names of the diseases, which were then translated by a local veterinarian.

## Breeding management

### Mating method, source of breeding bulls, and selection criteria

**Fig. 2:**
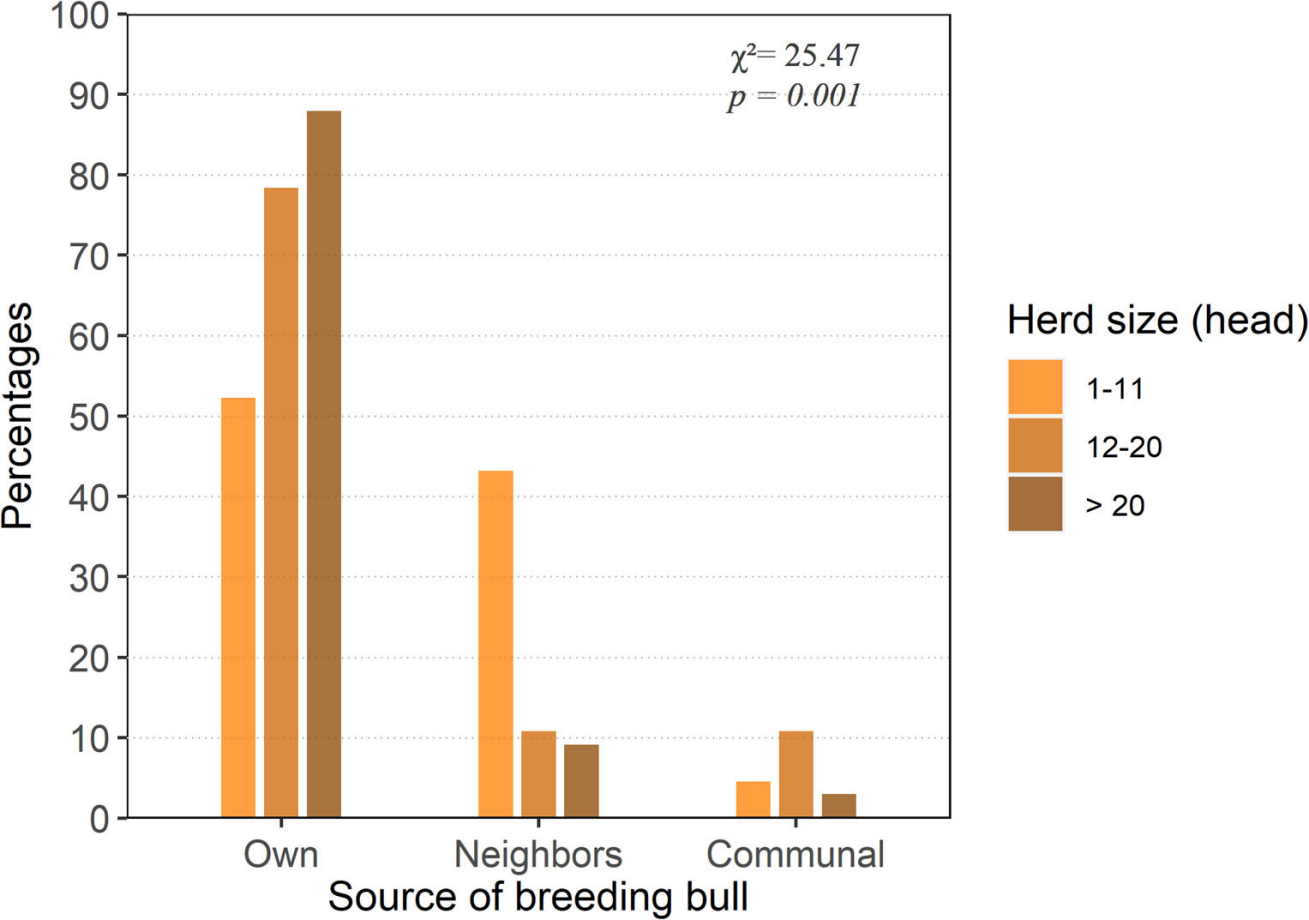
Source of breeding bulls across herd sizes

All farmers interviewed reported that natural mating was the only breeding method practiced since artificial insemination service using local bulls’ semen was not developed. However, the Sudani Center for Artificial Insemination and a Sudanese-Turkish Center for semen production provide services for cattle insemination in Sudan using semen from exotic bulls. The breeding bulls were allowed to run with the cows all year round. The majority of farmers (62.9%) selected the breeding bulls from their own herd, while 31.7% relied on bulls from their neighbors. Only 5.4% of the farmers relied on communal bulls, which were available at grazing sites. The source of breeding bulls was significantly associated with herd size (*χ*^2^ = 25.47, *p* = 0.001) (Fig. 2). Farmers who had small herd sizes (1–11 heads of cattle) were less likely to keep their own breeding bulls (residual = − 4.28, *p* = 0.0002); rather, they depended on their neighbors’ bulls (residual = 4.82, *p* = 0.00001). Conversely, farmers who had larger herd sizes (> 20 heads of cattle) tended to possess their own breeding bulls (residual = 3.25, *p* = 0.01) and were less likely to depend on neighbors’ bulls (residual = − 3.05, *p* = 0.02).

Regarding the selection criteria of breeding bulls, about 48.9% of farmers selected their bulls based on the observed phenotypes (e.g., coat color, width of chest, length of the body, scrotal circumference, or size of dewlap). The dark red coat color was preferred by the respondents, because dark red cattle were considered better milk producers, having heavier body weight. A similar proportion of farmers (48%) selected their bulls based on the observed milk yield of the bull’s dam while only a few of them (3.3%) based their selection on observed phenotypes (e.g., muscularity, daughters’ milk performance) of the bull’s sire. The average age of a breeding bull at selection was 25.6 ± 1.56 months, and the length of time for using bulls for mating was 5.9 ± 0.23 years.

### Animal recording

Without exception, none of the respondents reported keeping written records of production, reproduction, or animal pedigree. The main reason was that farmers claimed they had abilities to memorize all relevant information (81.7%). About 13.3% of the respondents had difficulties to keep written records. Accordingly, all information related to cattle production and reproduction characteristics, pedigree, and bulls’ selection criteria was based on farmers’ memories.

## Farm income and milk marketing

Nearly three-quarters (74.3%) of respondents indicated that they generated income from the sale of milk, followed by selling of live animals for slaughter (43.6%). Only 11.9% of farmers indicated that they derived their income from the sale of crops. About 48% of the farmers who sold milk indicated that they delivered the milk to final consumers using donkeys, followed by 27.3% who reported the use of cars or bicycles for milk delivery. The remaining 24.7% of the farmers sold their milk at the farm gate.

## Evaluation of adaptation and production traits

**Fig. 3:**
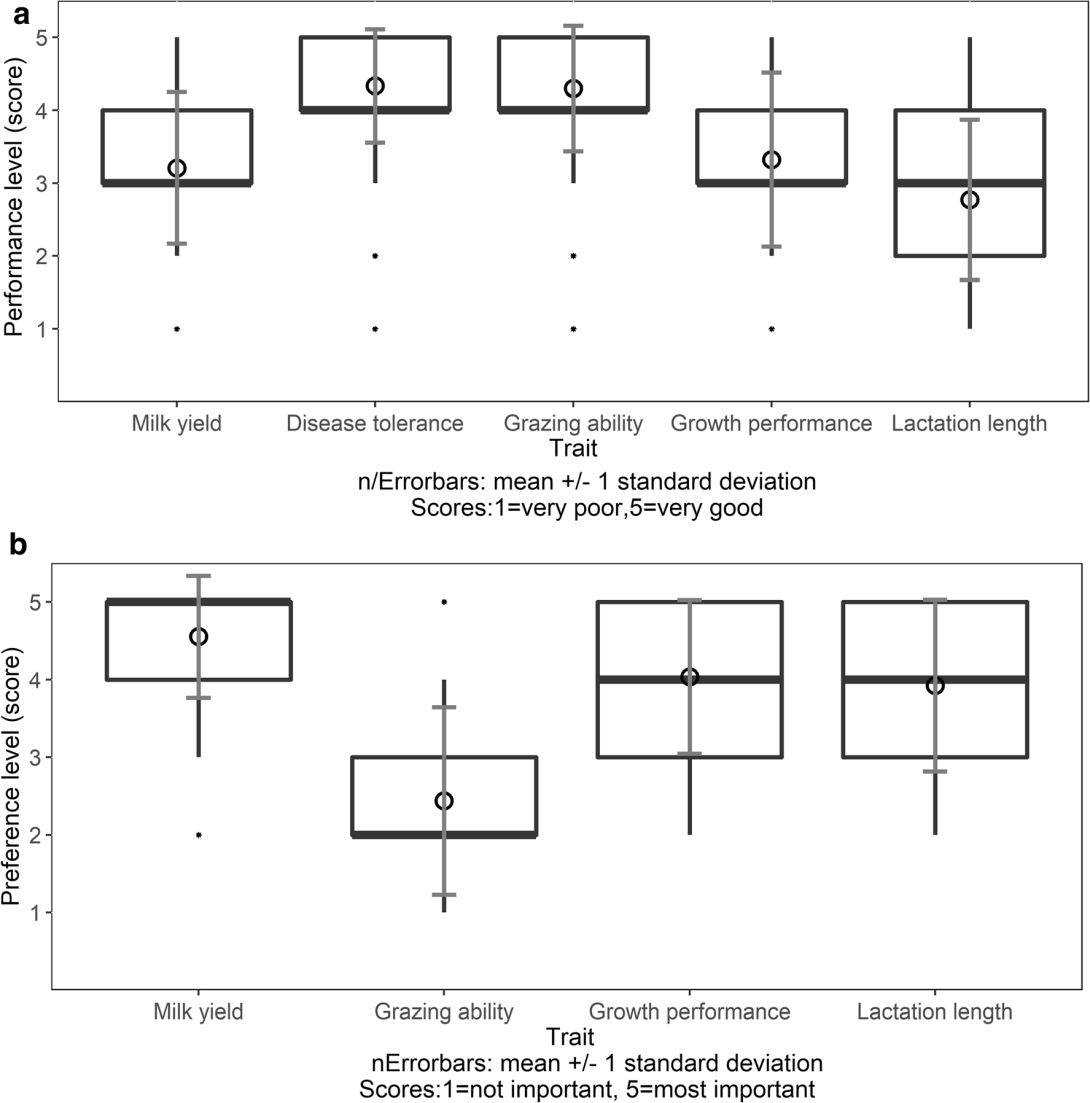
**a** Evaluation of trait performance and **b** preference for trait development

Evaluating trait performances of the Butana cattle breed, the highest scores were given to grazing ability (4.3 ± 0.86) and disease tolerance (4.3 ± 0.78), indicating that Butana cattle were able to walk long distances in search of pastures and watering points, and were disease tolerant (Fig. 3a). Moderate scores were found for growth performance (3.3 ± 1.19), milk yield (3.2 ± 1.04), and lactation length (2.8 ± 1.10). For future development of the traits in Butana cattle, farmers considered milk yield as very important (4.6 ± 0.79), followed by growth performance (4.0 ± 1.00), and lactation length (3.9 ± 1.12) (Fig. 3b). The grazing ability was rated less important (2.4 ± 1.21) for future development.

## Challenges of cattle production

Based on mean score values, respondents considered high costs of concentrate feeds (3.7 ± 0.10), lack of financial supports (3.7 ± 0.10), competition from crossbred dairy cattle in term of milk yield and selling price (3.7 ± 0.12), and lack of cattle improvement services (3.6 ± 0.08) as the major challenges associated with Butana cattle production (Table 4). Scarcity of rangeland was scored moderately important (3.4 ± 0.11). During the group discussions with the farmers, animal theft and high cost of water for watering cattle, specifically during the summer months, were reported as additional production challenges.

**Table 4:** Production challenges and their perceived importance

Item	Mean	SE
High cost of concentrate feed	3.72	0.10
Lack of financial support	3.69	0.10
Competition from crossbreds	3.68	0.12
Lack of cattle improvement services	3.57	0.08
Scarcity of rangeland	3.36	0.11

Number of respondents *n* = 202. *SE*, standard error

Scores (1 = not important, 2 = slightly important, 3 = moderately important, 4 = important, 5 = most important) indicate the importance given to the production challenges

## Farmers’ willingness to participate in a future dairy cattle improvement program

Table 5 summarizes the respondents’ willingness to participate in/adopt specific aspects associated with Butana cattle development in the future. The results revealed that almost all farmers (93%) were willing to exchange information, e.g., herd information. Only 26% of farmers indicated to be willing to keep written records. In general, highly educated farmers were 4.4 times more likely to adopt crossbreeding compared to lower educated farmers (*p* < 0.001) (Table 6). Highly educated farmers were 1.9 times more likely to adopt record keeping than farmers with a low education background (*p* < 0.05). On the contrary, the willingness to exchange information, establish farmers’ associations, keep written records, and exchange breeding bulls were not influenced by the farmer’s educational level.

**Table 5:** Willingness to adopt future development aspects

Item	Frequency	Percentage
Information exchange	187	92.3
Exchange of breeding bulls	135	66.8
Adoption of crossbreeding	137	67.8
Farmers association	129	64.0
Record keeping	52	25.7

Number of respondents *n* = 202

**Table 6:** Odds ratio estimates for impact of farmers’ education level (low vs high education and low education as the reference) on their willingness to participate in/perform relevant aspects for breed development

Development aspects	Odd ratio	95% CI	*p* value
Farmers’ association	1.41	0.77–2.60	0.26
Exchange of breeding bulls	0.58	0.33–1.06	0.08
Information sharing	0.81	0.33–1.92	0.46
Adoption of crossbreeding	4.43	2.14–9.20	0.001
Record keeping	1.92	0.28–0.99	0.047

*CI*, confidence interval

## Discussion

The development and successful implementation of a breeding program for local breeds require the definition of a comprehensive breeding objective, a holistic description of the production system, and the involvement of producers at every stage in the planning and implementation process (Kosgey et al. 2006; Duguma et al. 2010). In this study, the prevailing production conditions of the indigenous Butana cattle including farmers’ socio-economic characteristics, herd sizes and management, production objectives, farmers’ perception of trait performances, production challenges, and farmers’ willingness to participate in a future dairy cattle improvement program was described as a step towards the development of a community-based breeding program for the breed.

Our results showed that milk production is the main production objective for keeping Butana cattle. This is in line with the high proportion of respondents (74.3%) who considered the sale of milk as the main source of farm income. This result is consistent with the findings of Musa et al. (2006) who reported that owners of Butana cattle direct their production objectives towards increasing milk yield as a source of regular cash income and for home consumption. Accordingly, farmers prioritized milk yield over all other traits considered for future development of Butana cattle. High preferences for production traits such as milk yield, body size, or growth performance were also reported by farmers of Ankole cattle in Burundi, Rwanda, Tanzania, and Uganda (Wurzinger et al. 2006); Sahiwal cattle in Kenya (Ilatsia et al. 2012); and by dairy cattle farmers in Tanzania (Chawala et al. 2019). Farmers’ trait preferences play a crucial role in the development of breeding goals of sustainable livestock improvement breeding programs (Gemeda 2010; Getachew et al. 2010; Afras 2019). The importance of grazing ability for future development was medium, although the high costs of concentrate feeds and the scarcity of grazing land were major challenges. This indicates that under the prevailing production conditions of Butana cattle, grazing ability was considered optimal by the farmers; otherwise, they might be encouraged to move towards a more intensive system. In order to reduce the costs of concentrate feeds, these were usually only provided to crossbred animals and lactating cows during milking.

Selection criteria of breeding bulls reported in the present study are in accordance with previous findings. For instance, the milk yield of a bull’s dam (Musa et al. 2006) and general appearance of a breeding bull itself (Mohammed et al. 2014) were considered the most important criteria for bull selection by Butana cattle owners. Similarly, the selection of breeding bulls based on bull’s dam’s milk yield and body size or frame of the breeding bull itself was reported for Fulani cattle in Burkina Faso (Roessler 2019). However, the selection of breeding bulls in the absent of performance records as currently practiced by Butana cattle owners remains a challenge for genetic improvement of the breed. Noticeably, the average daily milk yield reported in the current study (6.6 kg) is consistent with previously reported estimates (6.9 kg) for Butana cows managed in a research station (Badri et al. 2011). However, the current report was based on farmers’ memories and may have been overestimated. In most dairy farming, a lactation length of 10 months is commonly accepted as a golden standard (Ayalew and Chanie 2018). However, a shorter lactation length of 6.7 months (201 days) was reported in our study, which is close to the mean lactation length estimates of 202.5 days and 208.6 days under field condition reported for the indigenous Kenana and Fuga cattle, respectively (Musa et al. 2006; Ibrahim et al. 2015). Calving interval, the time between two consecutive parturitions, is an important aspect of the reproductive efficiency of dairy cows and associated with economics of milk production (Ayalew and Chanie 2018; DeLay et al. 2020). The estimate of the calving interval for Butana cattle (13.7 months) in our study is much shorter than those reported for Butana (20.6 months), Kenana (17 months), and Fuga (15.9 months) cows under field conditions (Musa et al. 2006; Ibrahim et al. 2015). The differences may be due to differences in the herd management as well as breed differences, and for Butana farmers, the low calving interval may be promising and a strength of the breed. Nevertheless, the relatively high number of farmers keeping crossbred cattle and the strong willingness of farmers to adopt crossbreeding with exotic breeds emphasized farmers’ dissatisfaction with the current milk performance of the Butana breed. This finding supports previous claims about crossbreeding of local cattle with Holstein Friesian, which has been routinely adopted in Sudan in order to improve the milk performance of the indigenous breeds (Musa et al. 2008). According to the farmers in the present study, the main reason for keeping crossbred cattle was their higher milk production and market value. The complete absence of an organized or a formal breeding program for the Butana breed means that there are currently no alternative solutions to improve the breed’s performance genetically. This could be a catalyst for the increased willingness to adopt crossbreeding, which improves performance faster, at least in the short term. Crossbreeding potentially leads to the loss of important adaptation and disease resistance genes (Leroy et al. 2016; Sutarno and Setyawan 2015). Additionally, crossbreeding with the exotic breeds decreases purebred genetic diversity of indigenous breeds (Piyasatian and Kinghorn 2003) and can be seen as a threat to native genetic diversity (Hoffmann and Scherf 2006; Mwai et al. 2015).

The synergy between crop farming and cattle raising (mixed crop-livestock farming) reported by 89.6% Butana cattle farmers in the current study is a common practice found in many parts of Africa. For example, Ankole cattle in Burundi, Tanzania, Rwanda, and Uganda (Wurzinger et al. 2006), and Méré, Baoulé, and other cattle breeds in Côte d’Ivoire (Sokouri et al. 2014) are kept under mixed crop-livestock farming systems. The combination of cattle production with crop cultivation could be promising for overcoming the overall challenges of high costs associated with concentrate feeds and the general lack of financial support since farmers would be able to produce improved fodder or utilize crop by-products as low-cost feed resources.

Hired laborers were important for Butana cattle management, mainly for farmers who owned larger herd sizes and tend to move their cattle to grazing areas during the wet season, whereas family labor was more important for the farmers with small to medium herd sizes. Previous studies involving small dairy farmers in Burundi, Rwanda, Tanzania, Uganda, Ethiopia, and Burkina Faso have also reported that hired laborers and family members were important in raising indigenous cattle (Wurzinger et al. 2006; Zuria and Woredas 2009; Soudré et al. 2020). As most farmers owned small herd sizes and a simultaneously low number of breeding bulls, establishing farmers’ associations could be an important step for optimizing a future breeding program for the Butana cattle. Rege et al. (2011) emphasized that the selection and use of village sires in situations where herd sizes are small will require the cooperation of community members to record, select, and innovatively manage and use the selected sires. This could preserve genetic variability and keep the rate of inbreeding in the small herds at an optimal level, which are the main concerns in maintaining local breeds. That said, the exchange of breeding bulls that was generally accepted by the farmers in the present study may provide an opportunity for maintaining genetic diversity within the Butana cattle population.

Dairy production needs delivery of better animal health services and management. Even though the farmers in the current study indicated that Butana cattle are disease tolerant, the prevalence of diseases suggests that animal health services were poor and vaccination programs were lacking. The most important diseases reported in the current study (foot and mouth disease, trypanosomiasis, and mastitis) have also been reported in previous studies (Musa et al. 2006; Mohammed et al. 2014; Ahmed et al. 2016). In this regard, Wilson (2018) and FAO (2019) reported that vaccination programs in Sudan prioritized diseases affecting livestock exports, mainly contagious bovine pleuropneumonia and rinderpest, as foreign markets (e.g., United Arab Emirates, Jordan, Saudi Arabia, and Egypt) have more stringent health standards. Conversely, diseases that affect production such as mastitis and trypanosomiasis have received much less attention. Generally, crossbred animals are expected to be more prone to tropical diseases; however, in the current study, farmers did not distinguish between diseases along breed types.

Animal records are strongly required to establish genetic improvement programs and to support selection decisions in the long term. Our results revealed a complete absence of an official system for animal identification and performance recording. Instead, farmers purely relied on recalled memory. The absence of performance recording has also been reported in previous studies for other indigenous cattle breeds (Opoola et al. 2019; Roessler 2019). Major reasons associated with the lack of performance recording in most developing countries include the low awareness of potential benefits of recording to livestock owners, the research and development sector, and policy makers; problems of finding the right organization for animal recording; the challenge of attaining due participation; and insufficient technical know-how to implement and utilize records (Peters and Zumbach 2002). In the present study, farmers’ willingness to keep written records increased with higher educational level. Thus, educating farmers on the benefits of keeping written records could aid the adoption of record keeping. Such education can be offered to farmers collectively and with relative ease through novel farmers’ associations since the majority of the respondents were willing to establish this type of association. The establishment of farmers’ associations could enhance the strengthening of a so-called Butana cattle farmer lobby which represents and enforces farmers’ interests, mainly for solving problems associated with better access to financial support, as well as veterinary and breeding services (Buch et al. 2009; FAO 2013; Mueller et al. 2015; Ibeagha-Awemu et al. 2019; Lukuyu et al. 2019).

The present study highlighted the need for establishing community-based breeding programs for smallholder dairy cattle producers in Central Sudan. Our results showed that Butana cattle farmers mainly raised their animals for milk production. This was reinforced by farmers’ reliance on the sale of milk as the predominant source of income and the prioritization of milk yield as the most important trait for improvement. The willingness of farmers to engage in associations will be useful for the establishment of a community-based breeding program.
